# Extensive Lysine Methylation in Hyperthermophilic Crenarchaea: Potential Implications for Protein Stability and Recombinant Enzymes

**DOI:** 10.1155/2010/106341

**Published:** 2010-08-05

**Authors:** Catherine H. Botting, Paul Talbot, Sonia Paytubi, Malcolm F. White

**Affiliations:** ^1^Centre for Biomolecular Sciences, University of St Andrews, Fife KY16 9ST, UK; ^2^Departamento de Microbiologia, Facultat de Biologia, Universitat de Barcelona, Avenida. Diagonal 645, 08028 Barcelona, Spain

## Abstract

In eukarya and bacteria, lysine methylation is relatively rare and is catalysed by sequence-specific lysine methyltransferases that typically have only a single-protein target. Using RNA polymerase purified from the thermophilic crenarchaeum *Sulfolobus solfataricus*, we identified 21 methyllysines distributed across 9 subunits of the enzyme. The modified lysines were predominantly in *α*-helices and showed no conserved sequence context. A limited survey of the *Thermoproteus tenax* proteome revealed widespread modification with 52 methyllysines in 30 different proteins. These observations suggest the presence of an unusual lysine methyltransferase with relaxed specificity in the crenarchaea. Since lysine methylation is known to enhance protein thermostability, this may be an adaptation to a thermophilic lifestyle. The implications of this modification for studies and applications of recombinant crenarchaeal enzymes are discussed.

## 1. Introduction

Lysine methylation is found in all three domains of life. In bacteria, this posttranslational modification is restricted to a handful of ribosomal and flagellar proteins [1, 2]. In eukaryotes, lysine methylation is also restricted to a subset of proteins and catalysed by highly specific methyltransferases that can generate mono-, di-, and trimethylated lysines (reviewed in [3]). The most well-known examples are the histone proteins, where lysine methylation is carried out by sequence specific SET family methyltransferases using an S-adenosyl methionine (SAM) cofactor. These modifications result in changes in protein : protein interactions, chromatin structure and gene expression (reviewed in [4]). A limited number of other eukaryal proteins including notably the large subunit of Rubisco [5] are also subject to lysine methylation, though the function of these modifications is often not known [6]. More recently, proteome-wide studies of lysine methylation in the mouse brain [7] and *Saccharomyces cerevisiae* [8] have been added to the list of modified proteins. The latter study generated preliminary evidence for 25 monomethylated and 20 dimethylated lysines from a set of 2600 yeast proteins. The methylated proteins tended to have a higher abundance and longer half-life than average and included 11 ribosomal proteins [8]. 

The euryarchaeon *Methanosarcina mazei* encodes a clear SET-domain protein that has been shown to methylate a single lysine in the archaeal chromatin protein MC1, suggesting that mechanisms to modulate chromatin by posttranslational modification pre-date the divergence of the archaeal and eukaryal domains [9]. However, the distribution of this archaeal SET domain protein is limited to a few methanogens. Lysine methylation has also been noted in a handful of enzymes from the crenarchaeal Sulfolobales. Six methylated lysines were identified in glutamate dehydrogenase (GDH) from the hyperthermophile *Sulfolobus solfataricus* [10] leading the authors to speculate on a role in protein thermostability. Intriguingly, no lysine methylation was observed in the orthologous protein from the hyperthermophilic euryarchaeon *Pyrococcus furiosus* [11]. For *S. solfataricus* *β*-glycosidase, 5 lysines were methylated with an average of 3-4 modifications per protein [12]. The recombinant, unmodified protein purified from *Escherichia coli* showed an increase in denaturation and aggregation events compared to the native version, supporting the hypothesis that lysine methylation improves protein stability in extremophiles [12]. The extent of methylation of the *Sulfolobus* chromatin protein Sso7d was shown to increase with increasing growth temperature [13], again consistent with a potential role for methylation in thermostability. 

Since the limited evidence available suggested that lysine methylation in the crenarchaea might follow a different pattern to that observed in other lineages, we decided to investigate this phenomenon further by mass spectrometry. Here we show that lysine methylation is common in the crenarchaea and is catalysed by an unknown methyltransferase that lacks sequence specificity but is likely influenced by the local structure of the protein target. This has implications for the molecular biology of the crenarchaea, in particular the physical properties of crenarchaeal proteins and their recombinant counterparts.

## 2. Methods

### 2.1. Archaeal Biomass

*S. solfataricus* P2 biomass was a gift from Neil Raven (CAMR, Porton Down, UK). *Thermoproteus tenax* biomass was a gift from Bettina Siebers (University of Duisburg-Essen, Germany).

### 2.2. Purification of RNA Polymerase (RNAP)

RNAP was purified from *S. solfataricus* P2 biomass as described previously [14] by heparin and gel-filtration chromatography with the addition of an anion-exchange column (GE Healthcare MonoQ 5/5 column) as a final polishing step. Partly purified RNAP following gel filtration was loaded onto this column in buffer A (20 mM Tris-HCl pH 8.0, 50 mM NaCl, 1 mM DTT, 1 mM EDTA) and eluted with a linear gradient of buffer B (buffer A plus 1 M NaCl). Fractions containing pure RNAP were analysed by SDS-PAGE, pooled, and stored until required.

### 2.3. Mass Spectrometry

The purified RNA polymerase complex (5 *μ*L, 10 pmoles/*μ*L) was dialysed into 50 mM ammonium bicarbonate pH 8.0 using a membrane filter (Millipore, Billerica, MA) and trypsin (0.5 *μ*L, 0.1 *μ*g, Promega, Madison, WI) or clostripain (ArgC) (0.5 *μ*L, 0.1 *μ*g, Promega) was added. The samples were incubated at 37°C overnight.

After acidification (0.1% trifluoroacetic acid (TFA)), the peptides were separated using a Dionex UltiMate 3000 nanoLC (Dionex, Sunnyvale, CA) equipped with a PepMap100 C18 300 *μ*m × 5 mm trap and 75 *μ*m × 15 cm column (Dionex), using a 3.5 hr gradient of increasing acetonitrile concentration, containing 0.05% TFA (5%–35% acetonitrile in 3 hours, 35%–50% in a further 30 minutes, followed by 95% acetonitrile to clean the column). The eluent was spotted onto a MALDI target plate, along with *α*-cyano-4-hydroxycinnamic acid matrix solution (2 mg/mL in 70% acetonitrile, 0.1% TFA matrix solution) using a Dionex Probot spotter.

The nLC-MALDI MSMS runs were analysed using an Applied Biosystems 4800 MALDI TOF/TOF Analyser (Applied Biosystems, Foster City, CA) equipped with a Nd:YAG 355 nm laser in a plate-wide-data dependent manner. All spots were initially analysed in positive MS mode in the range 800 to 4000 m/z by averaging 1000 laser spots. The MS ions that satisfied the precursor criteria (200 ppm fraction-to-fraction precursor exclusion, S/N ratio >20) were selected for subsequent MSMS from the spot where the MS ion gave the highest counts, with up to 5 MSMS being acquired from each spot, selecting the strongest precursor ion first. MSMS spectra were acquired with a maximum of 3000 laser shots or until the accumulated spectrum reached an S/N ratio of 35 for 10 peaks. All MSMS data were acquired using 1 keV collision energy.

Peak lists were extracted from the MSMS spectra and analysed using the Mascot 2.1 search engine (Matrix Science, London, UK), against a database containing the* S. solfataricus *proteome. The data were searched with tolerances of 100 ppm for the precursor ions and 0.5 Da for the fragment ions, trypsin as the cleavage enzyme, assuming up to one missed cleavage, carbamidomethyl modification of cysteines as a fixed modification and mono and dimethylation of lysine, acetylation and methionine oxidation selected as a variable modification. The matches were then manually validated.

For ESI analysis, the acidified digested sample was separated using an UltiMate nanoLC (Dionex) equipped with a PepMap C18 trap and column, using a 210-minute gradient of increasing acetonitrile concentration, containing 0.1% formic acid (5%–35% acetonitrile in 180 min, 35%–50% in a further 30 minutes, followed by 95% acetonitrile to clean the column). The eluent was sprayed into a Q-Star XL tandem mass spectrometer (Applied Biosystems) and analysed in Information-Dependent Acquisition (IDA) mode, performing 1 second of MS followed by 3 seconds of MSMS analyses of the 2 most intense peaks seen by MS. These masses are then excluded from analysis for the next 60 seconds. MS/MS data for doubly and triply charged precursor ions were converted to centroid data, without smoothing, using the Analyst QS1.1 mascot.dll data import filter with default settings. The MS/MS data file generated was analysed as above with tolerances of 0.2 Da for the precursor and fragment ions.

Specific components of the polymerase complex were analysed from SDS-PA gel bands. The gel bands were excised and cut into 1 mm cubes. These were then subjected to in-gel digestion, using a ProGest Investigator in-gel digestion robot (Genomic Solutions, Ann Arbor, MI) using standard protocols [15]. Briefly, the gel cubes were destained by washing with acetonitrile and subjected to reduction, with dithiothreitol, and alkylation, with iodoacetamide, before digestion with trypsin or clostripain at 37°C. The peptides were extracted with 10% formic acid and analysed as described above. For the nLC-ESI MSMS analysis, shorter gradients (60 or 90 minutes) were used. For MALDI analysis, the digest solution (0.5 *μ*L) was applied to the MALDI target along with *α*-cyano-4-hydroxycinnamic acid matrix (0.5 *μ*L, 10 mg/mL in 50 : 50 acetonitrile: 0.1% TFA) and 0.1% TFA (0.5 *μ*L) and allowed to dry. MALDI analysis was performed using the 4800 MALDI TOF/TOF Analyser. The spot was analysed in MS mode as described above, and then the most intense peptides (up to 15) were selected for MSMS analysis and acquired as described above. The combined MS and MSMS data were analysed as described above, using GPS Explorer (Applied Biosystems) to interface with the Mascot search engine.

*T. Tenax *samples were digested similarly in solution or from SDS-PA gel bands and analysed by nLC-ESI MSMS on the QStar XL as described above.

## 3. Results

### 3.1. Identification of Methylated Lysines in *S. solfataricus* RNA Polymerase

**Figure 1:**
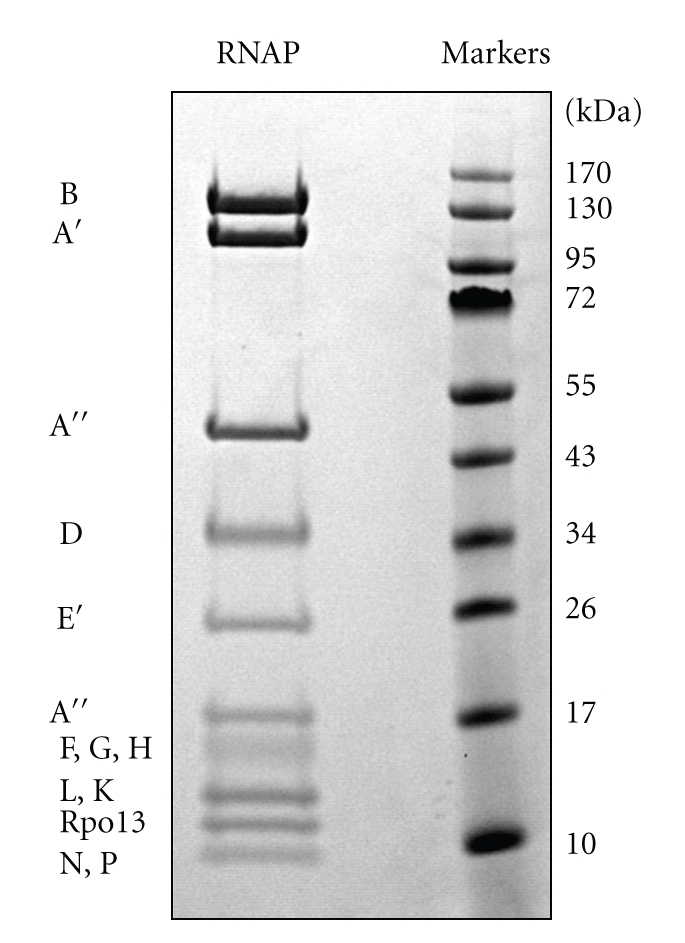
SDS-PAGE showing the purified RNA polymerase from *S. solfataricus*. In addition to all the expected subunits, a proteolytic product of the A′′ subunit was observed at 17 kDa.

We had previously purified archaeal RNA polymerase (RNAP), a complex enzyme consisting of 12-13 subunits, from *S. solfataricus* for transcription studies [14]. As crystal structures of RNAP are available [16, 17] and significant quantities of the protein could be purified, it represented an ideal system to investigate the extent of lysine methylation in this organism. RNAP was purified to homogeneity from 50 g of *S. solfataricus* strain P2 cells by sequential chromatography on heparin and Superdex 200 gel-filtration columns as described previously [14, 18]. A final chromatography step using a MonoQ column yielded pure RNAP as shown in Figure 1. 

RNAP was digested in solution using trypsin or clostripain (ArgC), and methylated peptides were identified by either LC-MALDI or nLC-ESI MS/MS. Subunits RpoD, E′, G, F, H, L, and K were also analysed individually by excising the relevant bands from a gel following SDS-PAGE and subjecting them to in-gel digestion and analysis by MALDI MS and MSMS or nLC-ESI MSMS. The Mascot search algorithm was used to identify potential sites of methylation. Subsequent manual validation centred particularly on whether the monomethyllysine immonium related ion at 98 m/z (immonium ion—17) and similarly the dimethyllysine immonium-related ion at 112 m/z were present, as well as assessing the confidence of the assignment of the specific site of modification [19]. Lysine methylation typically prevented cleavage by trypsin, providing further evidence of modification. In total, 21 monomethylated lysine residues were identified, from 9 of the RNAP subunits (Table 1, see Supplementary Table 1 at supplementary material available online at doi:10.1155/2010/106341). In several cases, the modified lysines were also detected in unmodified form, suggesting that the degree of methylation is not 100% and that a stochastic mixture of proteins with variable levels of methylation exists *in vivo*. In addition to lysine methylation, N-terminal peptides modified by acetylation were detected for four subunits: RpoA′ (Sso0225, N-terminal seq. SEKNIK), RpoB′ (Sso3254, ASNLTI), RpoD (Sso0071, SINLLH) and RpoF (Sso0751, SSVYIV). Only one unmodified N-terminal peptide was identified, that for RpoE′ (Sso0415, MYKLIK). These observations support previous work reporting acetylation of N-terminal serine and alanine residues in *S. solfataricus*, catalysed by the acetylase ssArd1 [20].

### 3.2. Sequence and Structural Context of Lysine Methylation

**Figure 2:**
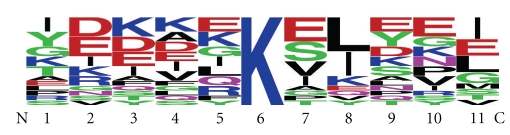
Sequence analysis of methylation sites in RNAP subunits. The frequency of each amino acid in an eleven-residue window centred on the modified lysine shows that there is no clear sequence specificity associated with methylation (generated by WebLogo [21]).

**Figure 3:**
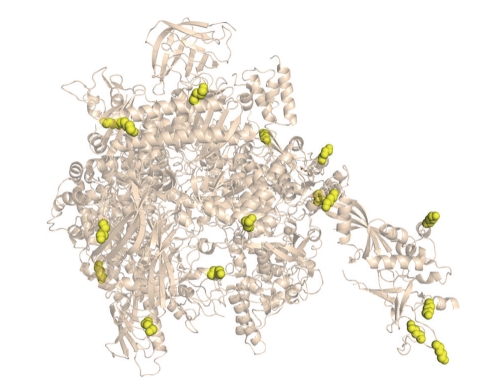
Mapping lysine methylation sites on the RNA polymerase crystal structure. The structure of RNAP from *S. shibatae* is shown [16] with methylated lysines listed in Table 1 indicated in yellow. This figure was generated using Pymol (The PyMOL Molecular Graphics System, Version 1.2r3pre, Schrödinger, LLC.)

**Figure 4:**
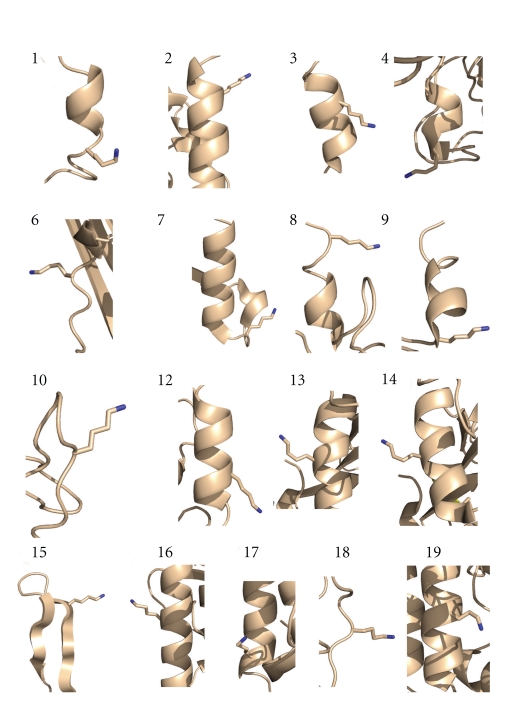
Structural context of methylation sites in RNAP subunits. Numbering is taken from Table 1. Fifteen of the sites are situated in *α*-helices whilst site 7 is in a hairpin turn and site 15 is situated in the *β*-sheet region of a *β*-hairpin. All structures are from *S. shibatae* RNAP structure (PDB 2WAQ) with the exception of 8, 9 and 10 which are from the *S. solfataricus* RNAP structure (PDB 2PMZ) as the two structures differ in this region of the E′ subunit.

The sequence context of methylation sites was analysed by plotting the frequency of each amino acid residue found at the 5 positions N- and C-terminal to the modified lysine (Figure 2). There was no detectable bias in amino acid residue or type (charged, hydrophobic, etc.) at any of the positions. This suggests that lysine methylation is catalysed by a methyltransferase that is largely sequence independent. To determine whether methylation occurred in a structural context, we examined the local structure of each of the 17 modified lysines visible in the RNAP crystal structures (Figure 3). All of the modified lysines were present on the surface of the protein and would be accessible to a modifying enzyme after protein folding had occurred. We noticed a striking correlation between structure and the site of modification, which was almost exclusively in a helical region of the protein (Figure 4). Exceptions were a lysine near the end of a *β*-turn in subunit RpoH and one in a hairpin turn in subunit RpoE′. Previous studies of lysines methylated in *S. solfataricus* *β*-glycosidase revealed two sites in *α*-helices and three in *β*-turns [12]. Together, these data suggest that lysine methylation in *S. solfataricus* is catalysed posttranslationally by a methyltransferase that is sequence independent but shows some structural specificity.

### 3.3. Lysine Methylation Is Prevalent in *Thermoproteus tenax*

Given the extensive methylation observed for RNAP subunits and *β*-glycosidase purified from *S. solfataricus* and the lack of any sequence dependence, lysine methylation in *S. solfataricus* is likely to be a general phenomenon involving many cellular proteins. To determine whether lysine methylation was specific to the *Sulfolobales* or more widespread, we investigated proteins from the crenarchaeon *Thermoproteus tenax*. In all, 90 proteins were represented by 3 or more tryptic peptides. Of these, 30 showed strong evidence for lysine methylation (52 sites in total, 6 of which were di-methylated; Supplementary Table 2) whilst about half the proteins showed no methylation and the remainder had weak or equivocal evidence for methylation. However, since the peptides identified did not provide complete sequence coverage, it is reasonable to assume that a proportion (and potentially a significant proportion) of these proteins do carry some lysine methylation *in vivo*. In other words, the incomplete peptide coverage leads to a potentially high false-negative rate but a low false-positive rate. These proteins had been partly purified from *T. tenax* for experiments aimed at detecting novel single-stranded DNA-binding proteins [22] and could be considered to represent a reasonably random sampling of the total soluble proteome. Although a bias towards more highly expressed proteins is likely, the methylated proteins have a wide variety of known or predicted functions. As with RNAP, there was no detectable sequence motif close to methylation sites. 

The widespread methylation observed from our limited sampling of these two species together with the absence of any sequence-specific modification leads us to predict that extensive lysine methylation may be a feature of the crenarchaea. In contrast, proteomic studies in the euryarchaea *Haloferax volcanii* [23], *Thermococcus gammatolerans* [24], *Pyrococcus furiosus* [25], and *Methanococcus maripaludis* [26] have not reported any evidence of extensive lysine methylation.

## 4. Discussion

The data presented here expand previous work on lysine methylation in the *Sulfolobales* and point to the existence of a novel sequence-independent lysine methylase that is distinct from the canonical SET-domain methylases prevalent in eukarya. Indeed, no SET-domain protein is detectable in any crenarchaeal genome using standard protein similarity searches. This enzyme appears to target lysines in alpha helices and near hairpin turns, suggesting that modification is posttranslational and has some structural specificity. It is of course possible that lysine methylation is catalysed by multiple enzymes rather than a single one. Our limited sampling of methylated proteins in *S. solfataricus* and *T. tenax* suggest that this modification may be quite prevalent in the crenarchaea, with a significant proportion of proteins carrying one or more methyllysine residues. It will be important to extend these studies to provide a more exhaustive survey of crenarchaeal proteomes. Whilst the data currently available for euryarchaea suggests that lysine methylation is not widespread, it will be interesting to determine whether this modification is also found in the thaumarchaea [27] or is an adaptation specific to a hyperthermophilic lifestyle.

Methylation of lysine increases the pKa of the side chain, allowing stronger ionic interactions to be made, and changes the hydropathy and solubility of proteins [12]. There are many examples in the literature where lysine methylation has resulted in improved stability of a variety of proteins and peptides. For example, methylation of human amyloid peptide *β*(25-35) reduced aggregation and toxicity [28]. Methylation of the protease bovine trypsin has been shown to increase the thermostability and reduce autolysis, resulting in a more useful enzyme for trypsinolysis [29]. Reductive (nonenzymatic) methylation is being used extensively in crystallography to improve crystallisability of proteins, a process that does not generally affect enzyme activity [30]. Our data suggest that crenarchaeal proteins expressed in recombinant systems may have altered physical properties due to a lack of lysine methylation compared to the situation *in vivo*. Potentially, this could affect their efficacy in biotechnological and other applications. The identification of the as-yet unknown crenarchaeal lysine methyltransferase, currently underway in our laboratory, could allow specific modification of lysine residues in a wide variety of proteins to improve stability and solubility.

## Supplementary Material

Supplementary Table 1 lists the experimental details including the quality of evidence for each methylated peptide identified in *Sulfolobus solfataricus* RNA polymerase.
Supplementary Table 2 lists the experimental details including the quality of evidence for each methylated peptide identified in proteins from *Thermoproteus tenax*.

## Figures and Tables

**Table 1:** Methylation sites identified in *S. solfataricus* RNA polymerase subunits^†^.

	Protein	Subunit	Residue	Peptide (methyllysine in bold)
1	Sso0225	RpoA′	395	**K**ELASTLAPGYIIER
2	Sso0225		659	**K**EIYNEIDR
3	Sso0227	RpoB′-C	12	IVE**K**TLYEMGVVPVEEVIR
4	Sso0227		311	GYKG**K**EYYR
5	Sso0227		349	FLQEF**K**ELSPEQAKR
6	Sso0071	RpoD	115	DI**K**SEDPSVVPISGDIPIVLLGTNQK
7	Sso0415	RpoE′	20	IPPNEFG**K**PLNEIALNELR
8	Sso0415		131	GIIFGE**K**SKKVIQKGDKVR
9	Sso0415		133	GIIFGEKS**K**KVIQKGDKVR
10	Sso0415		171	QPYLG**K**LEWITQTKK
11	Sso0415		179	LEWITQT**K**K
12	Sso0751	RpoF	54	CDAESAQ**K**VIEELSNIVSR
13	Sso0751		102	TYTSEDIQ**K**IIDIIR
14	Sso5468	RpoH	30	HEVLNIDEAY**K**ILK
15	Sso5468		68	**K**SQLYGEVVSYR
16	Sso5577	RpoL	71	DALL**K**AIENIR
17	Sso5577		88	GMTSHYIDEI**K**GLTK
18	Sso5865	RpoP	19	TFTDEQL**K**VLPGVR
19	Sso0396	Rpo13	66	**K**LFEDNYK
20	Sso0396		98	KAKKAVSKKVKKTKK**K**EKSVEG
21	Sso0396		100	KAKKAVSKKVKKTKKKE**K**SVEG

Monomethylated lysines are shown in bold. All lysines are conserved in the *S. shibatae* RNAP sequence with the exception of the lysine in peptide 19, which is an arginine in *S. shibatae* Rpo13.

**^†^**Supplementary Table 1 contains more detail and evidence supporting the identification of each modification site.
