# Supplementary material for: Extensive Lysine Methylation in Hyperthermophilic Crenarchaea: Potential Implications for Protein Stability and Recombinant Enzymes
Source: Archaea. 2010 Aug 5;2010:106341. doi: 10.1155/2010/106341 (PMC2929605; doi:10.1155/2010/106341)
Supplement: Supplementary file 1 — Supplementary Table 1 lists the experimental details including the quality of evidence for each methylated peptide identified in Sulfolobus solfataricus RNA polymerase. Supplementary Table 2 lists the experimental details including the quality of evidence for each methylated peptide identified in proteins from Thermoproteus tenax. [file 106341.f1.pdf]

Supplementary Table 1. Methylated lysines identified in *S. solfataricus* RNA polymerase

Protein	Enzyme	Method[a]	Peptide sequence	Ion Score	E value	Quality of evidence
RpoA' Rpo1N Sso0225	trypsin	Sol MALDI	**K**EIYNEIDR	63	2.4e-005	Good MSMS - b ion series covering site of modification and many y ions, specific immonium-17 ion
	ArgC	Sol MALDI	**K**ELASTLAPGYIIER	77	2.1e-007	Good MSMS - b ion series covering site of modification, specific immonium-17 ion
RpoB Rpo2 Sso0227	trypsin	Sol ESI	IVE**K**TLYEMGVVPVEEVIR	72	9.2e-006	Good MSMS with b ions either side of modification
	ArgC	Sol MALDI	GYKG**K**EYYR	42	0.0007	Reasonable MSMS spectrum - covers site of modification, specific immonium-17 ion
	ArgC	Sol MALDI	FLQEF**K**ELSPEQAKR	45	0.0005	Reasonable MSMS spectrum - no ions covering site of modification, specific immonium-17 ion
RpoD Rpo3 Sso0071	trypsin	Gel ESI	DI**K**SEDPSVVPISGDIPIVLLGTNQK	82	4.9e-08	No ions covering this part of peptide, so assuming increase in mass due to Me on K, but solid match to peptide sequence
RpoE' Rpo7 Sso0415	trypsin	Sol MALDI	LEWITQT**K**K	42	0.0028	Good MSMS covering site of modification, specific immonium-17 ion
	trypsin	Sol MALDI	IPPNEFG**K**PLNEIALNELR	124	1.6e-011	Good MSMS covering site of modification, specific immonium-17 ion
	ArgC	Sol MALDI	QPYLGKLEWITQT**K**K	100	1.2e-009	Good MSMS covering site of modification, specific immonium-17 ion
	ArgC	Sol MALDI	QPYLG**K**LEWITQT**K**K	40	0.0013	Reasonable MSMS, specific immonium-17 ion for 2 monomethylations
	ArgC	Sol MALDI	GIIFGEKS**K**KVIQKGDKVR	98	1.8e-009	Good MSMS covering sites of modification, specific immonium-17 ion
	ArgC	Sol MALDI	GIIFGE**K**S**K**KVIQKGDKVR	55	2.5e-005	can't distinguish which of adjacent K in 2 monomethylated peptide but specific immonium-17 ion for 2 monomethylated species

RpoF Rpo4 Sso0751	trypsin	Sol MALDI	TYTSEDIQ**K**IIDIIR	63	1.7e-005	Good MSMS covering site of modification, specific immonium-17 ion
	trypsin	Sol MALDI	CDAESAQ**K**VIEELSNIVSR	31	0.028	Poor MSMS – not many peaks, specific immonium-17 ion
RpoH Rpo5 Sso5468	trypsin	Sol ESI	HEVLNIDEAY**K**ILK	77	3.9e-006	Good MSMS, site of modification not covered
	ArgC	Sol MALDI	**K**SQLYGEVVSYR	101	8.5e-010	Good MSMS covering sites of modification, specific immonium-17 ion
RpoL Rpo11 Sso5577	trypsin	Gel ESI	DALL**K**AIENIR	58	5.8e-005	Good MSMS covering site of modification
	trypsin	Gel ESI	GMTSHYIDEI**K**GLTK	40	0.0043	Good MSMS covering site of modification
RpoP Rpo12 Sso5865	trypsin	Sol MALDI	TFTDEQL**K**VLPGVR	88	6.6e-008	Good MSMS covering site of modification, specific immonium-17 ion
Rpo13 Sso0396	ArgC	Sol MALDI	KAKKAVSKKVKKTKKKE**K**SVEG	110	2.7e-011	Good MSMS covering site of modification, specific immonium-17 ions
	ArgC	Sol MALDI	KAKKAVSKKVKKTKK**K**E**K**SVEG	63	1.4e-006	Good MSMS covering sites of modification, specific immonium-17 ions
	trypsin	Sol MALDI	**K**LFEDNYK	30	0.041	Reasonable MSMS - b1 ion covering site of modification plus specific immonium-17 ion

[a]Method abbreviations: Sol: solution digest; Gel: in-gel digest

Bold indicates site of monomethylation, Ion Score is the Mascot Ion Score, E value is the Mascot Expect value, which is a measure of the likelyhood that a match is purely random.

Supplementary table 2. Methylated lysines detected in *T. tenax* proteins

	Protein	Accession no.	Peptide seq	Ion Score	E value	Quality of evidence
1	rpoA2	TTX1676	GISL**K**DIEK	19	0.069	Reasonable MSMS – y ion accounting for site of modification. Unmodified peptide also present
			TLVEAAT**K**GDVEPFR	60	4.5 e-6	Good MSMS – y ion series covering site of modification
2	rpoD	TTX1672	ASATAVEEGS**K**AENV	38	0.00064	Good MSMS – y ion series covering site of modification
	-	TTX0308	QAFETD**K**YETVEK	37	0.0016	Good MSMS – doubly charged y ion series covering modification – unmodified peptide also present (Ion Score 49) spectra v similar
			FYDI**K**AK	34	0.0042	Good MSMS – y ion series covering site of modification– unmodified peptide also present (Ion Score 19) spectra v similar
			FYDIKAK	38	0.001	Good MSMS – y ion series covering site of modification
3	Ribosomal protein L30	TTX1409	**K**YGWNSFEELALAFVSGEIER	53	1.3 e-5	Good MSMS – b1 ion covering modification
			IVGDKPLTLDHL**KK**	23	0.0097	Reasonable MSMS – 1 site of modification covered, coverage of alternative site (K5) refutes modification at K5
4	hypothetical	TTX1876	EYTPEEIA**K**	41	0.00064	Good MSMS – y ion series covering modification – unmodified peptide also present (Ion Score 43)
			VAIV**K**AQLTR	21	0.032	Reasonable MSMS - unmodified peptide also present (Ion Score 17 (19=Ident)
5	Conserved	TTX0588	INVE**K**IVK	32	0.0045	Reasonable MSMS – b ion series covering mod

	hypothetical					
6	nusA	TTX1674	VT**K**SPSGNLVAITTVAPSDK	66	3.1 e-5	Good MSMS – unmodified peptide also present (Ion Score 51) and dimethylated - see below
			VTKSPSGNLVAITTVAPSDK	49	0.00056	Good MSMS
			SQAALAVG**K**GGNNVR	33	0.0042	Reasonable MSMS – covering site of modification - unmodified peptide also present (Ion Score 60)
7	Malate dehyrogenase	TTX1427	FQQSVEAIS**K**	52	6.7 e-7	Good MSMS
			IVLIDII**K**	28	0.0068	Reasonable MSMS
8	sdhB/frdB	TTX0863	ISVLDVLL**K**	25	0.018	Reasonable MSMS
9	ferredoxin	TTX0985	A**K**YGIQVDPLPPS	24	0.019	Reasonable MSMS
10	Conserved hypothetical	TTX1408	VSTGI**K**ELDEALEGGIPK	60	2.9 e-6	Good MSMS - unmodified peptide also present (Ion Score 72)
			**K**EPEAMPQIVVIDIFGLLK	53	7.7 e-7	Good MSMS - unmodified peptide also present (Ion Score 65)
			QLGMDFE**K**	28	0.008	So-so MSMS - unmodified peptide also present (Ion Score 43)
			KYSYQLK	28	0.017	So-so MSMS – immonium ion -17 present -unmodified peptide also present (Ion Score 28)
11	hypothetical	TTX1576	**K**LQQITSLLK	47	8.4 e-5	Good MSMS b ion series covering modification 2Me spectrum similar
			**K**LQQITSLL**K**	25	0.011	
			**K**WFFEEAVPR	44	0.00019	Good MSMS
			SELIT**K**GEK	26	0.15	Reasonable MSMS

			LA**K**EEVELLK (mono & di)	27	0.015	Mono and diMe – similar reasonable spectra
12	Putative dehydrogenase	TTX0535	AAALINYIDSIFV**K**<br><br>**K**VVESITQYLEGR (mono & di)	62<br><br>92 (m)<br>35 (di)	3 e-1.1<br><br>4.9 e-9<br>0.0016	Good MSMS<br><br>Good MSMS<br>Reasonable MSMS
13	hypothetical	TTX1565	DVEEELS**K**LPVDVSR	34	0.0014	Good MSMS – y ion series covering modification
14	Conserved hypothetical	TTX2090	ALGAEVEQL**K**<br><br>EVAGAVI**K**EGDLSEAPVAVQFK	34<br><br>27	0.0025<br><br>0.0029	Reasonable MSMS - unmodified peptide also present (Ion Score 14)<br>Reasonable MSMS
15	Conserved hypothetical	TTX1155	TGAPIYVADTLGSLTEDASQYIQPPSG**K**	73	7.2 e-8	Good MSMS
16	porA	TTX1785	GIIGAIDAEYG**K**<br>AI**K**GEYGDWIVLL	45<br>25	0.00022<br>0.011	Good MSMS<br>Reasonable MSMS
17	Gsp putative type II secretion system protein	TTX0896	AELLQALAD**K**GVIDAEAVAEAVR	59	1.7 e-6	Good MSMS
18	Rfc small subunit	TTX1851	I**K**LTEDGIDAIYEISQGDMR	30	0.002	Reasonable MSMS
19	Eif-1A	TTX0636	GYGEVIDNLD**K**LA	46	0.00011	Good MSMS – y ion series covering modification
20	Conserved hypothetical	TTX2059	LSAILV**K**	24	0.033	So-so MSMS
21	aEF-1 alpha subunit	TTX0013	TGQTIEQ**K**PQFIK<br><br>TGQTIEQKPQFIK	42<br><br>19	0.0005<br><br>0.068	Good MSMS – covering site of modification<br><br>Reasonable MSMS

			TIAAGQIVEIKPA**K**VEIK	35	0.00093	reasonable MSMS – covering site of modification
			VETGVIKPGD**K**VIVMPPAK	32	0.0026	reasonable MSMS – covering site of modification
22	Conserved hypothetical	TTX0298	**K**AVEYGLSLGAK	23	0.038	Reasonable MSMS
23	lysS	TTX2056	L**K**ELQVPLK	35	0.0015	Reasonable MSMS
24	Conserved hypothetical	TTX0105	VLDMDELE**K**YR	33	0.0037	Good MSMS – covering site of modification
25	Protein-L-isoaspartate O-methyltransferase	TTX0958	E**K**LAYVSFVK	23	0.02	Reasonable MSMS- unmodified peptide also present (Ion Score 27)
26	NAD dependent dehydrogenase	TTX1281	SLG**K**NELETATGER	38	0.00061	Reasonable MSMS- unmodified peptide also present (Ion Score 42)
27	Hypothetical protein	TTX1885	QDAADQVV**K**MLDQPVER	59	3.6 e-6	Good MSMS – covering site of modification
28	Putative DNA helicase	TTX0530	EIA**K**LPGFGEK	31	0.0073	Good MSMS – covering site of modification - unmodified peptide also present (Ion Score 36)
			LIDASSAA<u>K</u>LAAEIESTSASR	50	3.3 e-5	Good y ion series up to site of modification, doubly charged y ions N-term to modification
29	Conserved hypothetical	TTX1840	LEDVL**K**ELA	30	0.007	Reasonable MSMS – covering site of modification - unmodified peptide also present (Ion Score 36)
			QEEIL**K**SVADLNR	30	0.0045	Reasonable MSMS
30	Conserved hypothetical	TTX1724	LVPSE**K**IGTPGVDGSIAIYETK	26	0.0046	Reasonable MSMS

Bold indicates monomethylation of lysine, also (m), Underlining indicates dimethylation of lysine, also (di), Ion Score is the Mascot Ion Score, E value is the Mascot Expect value, which is a measure of the likelyhood that a match is purely random.
